# HEALTH CARE RESEARCH AND PROVISION AMONG OLDER ADULT GROUPS HISTORICALLY UNDERREPRESENTED IN RESEARCH

**DOI:** 10.1093/geroni/igae098.1922

**Published:** 2024-12-31

**Authors:** Aneeka Ratnayake, Mark Brennan-Ing

**Affiliations:** Chair; Discussant

## Abstract

In this symposium we will discuss research focusing on minority populations who are often under-represented in research. We will focus on research among three groups broadly: racial and ethnic minority groups, sexual and gender minority groups, and patients in low- and middle-income countries. Our first presentation will discuss enrollment of racial and ethnic minority groups in the All of Us Research Program—a program aimed at oversampling those under-represented in research in the United States. The generalizability of those living with HIV captured in the All of Us database will be ascertained as compared to the overall population living with HIV in the United States. The second presentation will discuss whether issues of potentially inappropriate medication prescriptions are different for sexual and gender minority older adults compared to cisgender heterosexual older adults. In this presentation, disparities in potentially inappropriate medication prescriptions among this population will be highlighted. The final presentation will discuss correlates of physical activity among older adults with and without HIV in Uganda. In this presentation the contextual differences for studying physical activity among older adults in the Global South will be discussed. Together, these presentations highlight the need to consider vulnerabilities of populations under-represented in research and especially in older adults. Though specific emphasis will be placed on three under-represented groups, the overarching goal of this symposium is to emphasize the impact of prioritizing representation, intersectionality, and identity when conducting gerontological research and providing healthcare to older adults. HIV, AIDS and Older Adults Interest Group Sponsored Symposium

